# Literary Notes

**Published:** 1908-06

**Authors:** 


### LITERARY NOTES.

Messrs. W. B. Saunders Company, medical publishers of Philadelphia and London, announce for publication before June 30, 1908, a list of books of unusual interest to the profession, calling special attention to the following: Bandler's Medical Gynecology, treating exclusively of the medical side of this subject; Bonney's Tuberculosis; Volume II, Kelly and Noble's Gynecology and Abdominal Surgery; Volume IV, Keen's Surgery; Gant's Constipation and Intestinal Obstruction; Schamberg's Diseases of the Skin and the Eruptive Fevers; John C. DaCosta, Jr's., Physical Diagnosis; Todd's Clinical Diagnosis; Camac's Epoch-making Contributions in Medicine and Surgery. All these works will be profusely illustrated with original pictures.

The Boston Medical and Surgical Journal dedicated its issue of May 7, 1908, to Reginald Heber Fitz, M.D., L.LD., Hersey Professor of the Theory and Practice of Physic in Harvard University in honor of his sixty-fifth birthday, May 5, 1908. It is a handsome number of 148 pages contributed by his former house pupils at the Massachusetts General Hospital and Assistants in the Department of the Theory and Practice of Physic at Harvard University. A handsome photogravure of Dr. Fitz appears as a frontispiece and the entire number is filled with articles of scientific value. It is a credit to all concerned.

